# Neural correlates of Korean proverb processing: A functional magnetic resonance imaging study

**DOI:** 10.1002/brb3.829

**Published:** 2017-09-12

**Authors:** You Gyoung Yi, Dae Yul Kim, Woo Hyun Shim, Joo Young Oh, Sung Hyun Kim, Ho Sung Kim

**Affiliations:** ^1^ Department of Rehabilitation Medicine Seoul National University Hospital Jongno‐gu, Seoul Korea; ^2^ Department of Rehabilitation Medicine Asan Medical Center Songpa‐gu Seoul Korea; ^3^ Department of Radiology and Research Institute of Radiology University of Ulsan College of Medicine Korea; ^4^ Asan Institute for Life Science Asan Medical Center University of Ulsan College of Medicine Seoul Korea

**Keywords:** figurative language, functional magnetic resonance imaging, Korean proverb, right hemisphere

## Abstract

**Introduction:**

The Korean language is based on a syntactic system that is different from other languages. This study investigated the processing area of the Korean proverb in comparison with the literal sentence using functional magnetic resonance imaging. In addition, the effect of opacity and transparency of proverbs on the activation pattern, when familiarity is set to the same condition, was also examined.

**Methods:**

The experimental stimuli included 36 proverbs and 18 literal sentences. A cohort of 15 healthy participants silently read each sentence for 6 s. A total of 18 opaque proverbs, 18 transparent proverbs, and 18 literal sentences were presented pseudo‐randomly in one of three predesigned sequences.

**Results:**

Compared with the literal sentences, a significant activation pattern was observed in the left hemisphere, including the left inferior frontal gyrus, in association with the proverbs. Compared with the transparent proverbs, opaque proverbs elicited more activation in the right supramarginal gyrus and precuneus.

**Conclusions:**

Our study confirmed that the left inferior frontal gyrus mediates the retrieval and/or selection of semantic knowledge in the Korean language. The present findings indicated that the right precuneus and the right supramarginal gyrus may be involved in abstract language processing.

## 1 INTRODUCTION

Nonliteral language includes proverb, metaphor, idiom, ironic expression, and metonymy. The metaphor is the extension of literal meaning using a more abstract representation (Zempleni, Haverkort, Renken, & Stowe, 2007). Idioms, the most common forms of figurative language, may refer to frozen metaphors, but are fixed and familiar expressions (Zempleni et al., 2007). A proverb is a short sentence that people often quote based on practical experience or common sense, which is sometimes metaphorical.

In studies of metaphor comprehension, perhaps the most crucially debated issue is whether the right hemisphere (RH) contributes to the interpretation of the nonliteral language. To date, a few studies have reported a crucial role of the RH in interpreting metaphors (Anaki, Faust, & Kravetz, 1998; Bottini et al., 1994; Schmidt & Seger, 2009). In neuropsychology, it is widely accepted that the RH plays a special role in the processing of idiomatic expressions (Van Lancker & Kempler, 1987).

However, other studies have reported that the left hemisphere (LH), but not the RH, plays a special role in idiom comprehension (Oliveri, Romero, & Papagno, 2004; Rapp, Leube, Erb, Grodd, & Kircher, 2004). Rapp et al. (2004) suggested that the RH theory of metaphor comprehension should be critically reevaluated because reading metaphors, compared with literal sentences, resulted in signal changes in the left lateral inferior frontal and temporal gyri, as well as the left posterior middle/inferior temporal gyri. Oliveri et al. (2004) reported that the comprehension of opaque idioms is closely related to the activity of temporal areas of the LH, which is similar to the processing of literal sentences. They also suggested that the left superior temporal cortex plays a crucial role in the process of idiom comprehension.

However, recent studies suggested that the contrast between literal sentences and metaphors is not limited to the RH or LH, but is instead bilateral (Lauro, Tettamanti, Cappa, & Papagno, 2008; Zempleni et al., 2007). Zempleni et al. (2007) reported that figurative sentences activate the bilateral inferior frontal and middle temporal gyri, suggesting that figurative language comprehension is more complex than literal sentence interpretation in both the LH and RH language areas and their homologs (Zempleni et al., 2007). Similarly, Lauro et al. (2008) reported that, during idiomatic interpretation, the bilateral medial prefrontal area significantly increased the connection between frontotemporal areas, which indicates an important role for the prefrontal cortex in the comprehension of idioms.

Different neural activation patterns have been reported based on familiarity, figurativeness, and novelty (Mashal, Faust, & Hendler, 2005; Rapp et al., 2004; Van Lancker & Kempler, 1987). In addition, it has been reported that the activated regions are different depending on the transparency/opacity of the language (Oliveri et al., 2004; Proverbio, Crotti, Zani, & Adorni, 2009). Although predictability is important for initiating the processing, transparency is also a factor affecting idiom perception (Sela, Panzer, & Lavidor, 2017). The figurative meaning of opaque proverbs is very different from the literal meaning, and it generally includes social and cultural aspects. According to a recent study on Chinese idioms (Yang et al., 2016), the opaque idiom resulted in a stronger activation in the right parietal area.

To our knowledge, there are no reports on the brain regions activated during Korean proverbs comprehension. The Korean language has a syntactic system different from other languages. Hangul (Korean language) differs from Chinese characters in that each letter alone has no meaning, but the combination of different letters, like the alphabet, becomes meaningful. However, the difference with the alphabet is that Hangul consists of a combination of initial, middle, and sometimes end strokes. Proverbs in Korean are similar to other languages in that they are very familiar and descendant. However, they may have a literal or a hidden meaning. This study investigated the processing area of the Korean proverb in comparison with the literal sentence using functional magnetic resonance imaging (fMRI). Furthermore, we hypothesized that proverb processing may be affected by the transparency/opacity of the language also in fMRI experiments, with a specific focus on the interpretation of proverbs in Korean when familiarity is set to the same level.

## 2 PARTICIPANTS AND METHODS

### 2.1 Participants

We prospectively recruited 15 healthy adults (seven men; eight women) with a mean age of 30.2 years (range: 27–33 years old). The inclusion criteria were as follows: (1) right handed by self‐reporting; (2) native Korean speakers; (3) no previous history of neurological or psychiatric disease; and (4) an education level corresponding to at least 1 year of college. Participants who were contraindicated for fMRI, such as individuals with metallic implants, claustrophobia, or a history or risk of seizure, were excluded. All participants provided written informed consent. The protocol of the study was approved by the institutional review board of the Asan Medical Center.

### 2.2 Types of figurative language

Figurative language includes idioms, metaphors, and irony and is nonliteral (Schmidt & Seger, 2009). It is not a unitary class because it includes language with various characteristics, including literality (whether or not a phrase also has a literal meaning), familiarity, and opacity/transparency (whether or not the meaning can be derived from the triggered image) (Glucksberg, Brown, & McGlone, 1993). Given that figurative language is not a homogeneous classification, we categorized proverbs by familiarity and opacity/transparency.

### 2.3 Transparency

The term transparency describes whether or not a meaning can be elicited from the evoked image (Glucksberg et al., 1993). Some types of proverbs are highly transparent, whereas others are nontransparent; thus, the metaphorical interpretation is difficult to make without knowing the figurative language. To identify the effects of transparency processing in proverb comprehension, we compared opaque and transparent proverbs. For objective measurement of the transparency of each stimulus, 200 proverbs were categorized by two speech‐language pathologists, a scholar of Korean literature, and five participants selected from the population representative of the sample that later underwent the fMRI study to evaluate the semantic distance.

### 2.4 Familiarity

Familiarity indicates whether an individual “knows” an expression (Rapp, Mutschler, & Erb, 2012). Familiar proverbs are easier to understand than unfamiliar proverbs (Nippold & Haq, 1996). Nippold and Haq (1996) proposed the “language experience” hypothesis, which holds the view that comprehension develops via meaningful exposure to figurative sentences, thereby facilitating easy understanding. The degree of familiarity of 200 proverbs (100 transparent proverbs and 100 opaque proverbs) was categorized by 100 native Korean speakers using scores ranging from 1 to 5 (1 = never used or encountered; 5 = frequently used/encountered).

The two proverb groups for finally selected 54 proverbs were scored similarly; opaque proverbs had a mean score of 4.04 (standard deviation [*SD*] = 0.47), and transparent proverbs had a mean score of 4.13 (*SD* = 0.62). To ensure that the participants understood the instructions, 15 participants evaluated the experimental stimuli after the data acquisition as a part of the study.

### 2.5 Stimuli

The final experimental stimuli for the participants consisted of (1) 54 opaque proverbs, (2) 54 transparent proverbs, and (3) 54 literal sentences. Table 1 summarizes the characteristics of each type of proverb (opaque and transparent) and literal sentences and Table 2 describes the examples of each type of proverb (opaque and transparent) and literal sentences.

**Table 1:** characteristics of the final experimental stimuli

Condition	Semantic distance	Proverb status	Familiarity	Number of Korean characters
Opaque proverbs	5.02 (0.63)	1 (0)	4.04 (0.47)	11.19 (2.63)
Transparent proverbs	2.33 (0.48)	1 (0)	4.13 (0.62)	11.26 (2.47)
Literal sentences	0.02 (0.14)	0 (0)	4.20 (0.56)	11.15 (2.48)

**Table 2:** Example of literal sentence, opaque proverb, and transparent proverb

Classification	Korean	Alphabet sound	Meaning
Literal sentence	마라톤 선수가 결승점에 도착했다	Ma/la/ton seon/su/ga gyeol/seung/jeom/e do/chag/haess/da	The marathoner has arrived at the finish line
Opaque proverb	병에 찬 물은 저어도 소리가 안 난다	Byeong/e chan mul/eun jeo/eo/do so/li/ga an nan/da	Bottled water does not sound even when poured. Hidden meaning: A person who actually knows a lot is humble and does not pretend to know
Transparent proverb	가는 말이 고와야 오는 말이 곱다	Ga/neun mal/i go/wa/ya o/neun mal/i gob/da	If the word you say is good, then the word coming back at you is good

**Figure 1:**
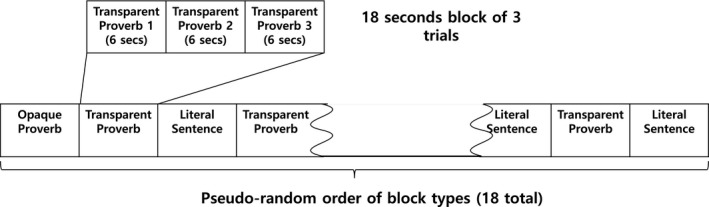
Experimental Design. For each trial, a sentence appeared in the center of the screen for 6 s. The 54 sentences were randomly assigned to 18‐s blocks of three sentences of the same type. Blocks were arranged in a pseudo‐random order. There were 18 blocks that consisted of six blocks of transparent figurative proverbs, six blocks of opaque proverbs, and six blocks of literal sentences

For each participant, a total of 36 proverbs and 18 literal sentences were presented pseudo‐randomly in one of three predesigned sequences (Figure 1). All sentences were presented on a screen while the participant was lying inside the MRI scanner. The regions activated in response to the different stimuli were compared as followed: (1) literal with proverbs, (2) transparent with opaque proverbs, and (3) familiar with unfamiliar proverbs.

### 2.6 Acquisition of the MRI data

The MRI data were acquired with a 3.0 T system (Achieva; Philips Medical Systems, Best, the Netherlands) using an eight‐channel sensitivity‐encoding head coil. For functional imaging, blood oxygen level–dependent (BOLD) contrast images were measured by single‐shot gradient‐echo/echo‐planar imaging (GE/EPI) with the following parameters: repetition time/echo time (TR/TE), 3,000/35 ms; flip angle, 90°; field of view, 220 mm; matrix, 128 × 128; and slice thickness, 4 mm with no gap. A high‐resolution anatomical three‐dimensional (3D) volume image was also obtained using a 3D gradient‐echo T1‐weighted sequence with the following parameters: TR/TE, 9.9/4.6 ms; flip angle, 8°; field of view, 224 mm; matrix, 448 × 448; and slice thickness, 1 mm with no gap.

Sentences were presented visually by rear projection to a screen that was mounted inside of the bore. Sentences were shown through a mirror that was mounted on the head coil. The first four initial dummy scans were discarded before the fMRI task to allow for steady‐stage magnetization. The E‐Prime software (Psychology Software Tools, Pittsburgh, PA, USA) was used to generate all design and control stimuli in the scanner.

### 2.7 Data analysis

Data were processed using the FSL 5.0.6 software package (Oxford Centre for Functional Magnetic Resonance Imaging of the Brain [FMRIB] Analysis Group, RRID: SCR_002823, www.fmrib.ox.ac.uk/fsl). Skull stripping and nonbrain tissue removal were performed with a Brain Extraction Tool (BET). Head motions were corrected with Motion Correction using FMRIB's Linear Image Registration Tool (MCFLIRT) along with a Gaussian kernel of 5‐mm full‐width half maximum that used spatial smoothing. All fMRI sessions were analyzed using general linear modeling with both the lower‐ and higher‐level modes for the fMRI Expert Analysis Tool (FEAT). All fMRI data were registered to the anatomical *T*1 volumes for each participant for registration to the standard space (MNI‐152 space). All transformations were carried out using a 12 Degrees of freedom linear transformation. Statistically based parametric images were generated using a significance threshold of uncorrected *p* < .001, uncorrected for multiple comparisons, which corresponded to a *T*‐value threshold of 4.5 that is commonly used in fMRI studies (Badre & D'Esposito, 2007).

## 3 RESULTS

### 3.1 Behavioral data

The task was to read the sentences, and the feedback was verbally reported after the fMRI scan. All participants reported that they had concentrated on all proverbs and literal sentences.

### 3.2 Proverbs versus literal sentences

**Figure 2:**
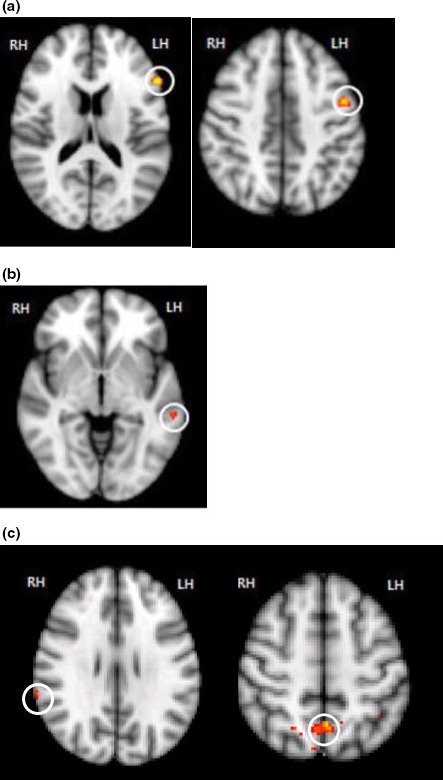
Activations for proverbs compared with a literal sentence. The images show regions with significant differences in activation (*p* < .001, uncorrected [*T* ≥ 3.30]) and the spatial extent of activation (kE) was ≥10 voxels. (a) Proverb > Literal sentence, left inferior frontal gyrus, left precentral gyrus; (b) Transparent proverb > opaque proverb, left middle temporal gyrus; (c) Opaque proverb > Transparent proverb, right supramarginal gyrus, right precuneus

As described above, we categorized the proverbs by opacity/transparency. When compared with a literal sentence, a significant activation pattern in the LH was observed in response to proverbs from each of the two categories (Table 3, Figure 2, *p* < .001).

**Table 3:** Anatomical regions that showed significant differences compared with literal sentences

kE	*T*	Lat	Anatomical region	*x*	*y*	*z*	BA
Proverbs > Literal sentences							
34	3.41	LH	Inferior frontal gyrus	−58	22	16	45
13	3.34	LH	Precentral gyrus	−47	−2	44	6
14	3.52	RH	Precuneus	2	−60	52	7
Transparent proverbs > Literal sentences							
12	3.42	LH	Inferior frontal gyrus	−58	24	16	45
16	3.34	LH	Precentral gyrus	−48	−2	44	6
12	3.45	LH	Middle temporal gyrus	−60	−34	−4	21
Opaque proverbs > Literal sentences							
10	3.45	LH	Inferior frontal gyrus	−58	22	16	45
12	3.42	RH	Supramarginal gyrus	60	−38	28	40
36	3.56	RH	Precuneus	2	−60	52	7

L, left; R, right; H, hemisphere.

All areas reported were significant at *p* uncorrected ≤.001 (*T* ≥ 3.30), the spatial extent of activation (kE) was ≥10 voxels. Areas are presented with the stereotactic coordinates according to Talairach and Tournoux (*x*,* y*,* z*; 1998), the cytoarchitectural designation according to Brodmann (BA), the maximum *T* value (*T*), and the extent (kE) of the activated clusters. In order to emphasize laterality effects, the hemispheric lateralization (Lat) is presented in a separate column.

Compared with literal sentences, transparent proverbs elicited activation in the left inferior frontal gyrus (IFG), left middle temporal gyrus (MTG), and precentral gyrus. Additionally, compared with literal sentences, opaque proverbs elicited activation in the left IFG and right supramarginal gyrus (SMG).

### 3.3 Opaque proverbs versus transparent proverbs

Opaque proverbs versus transparent proverbs elicited significantly increased the activation of the right SMG (Table 4, Figure 2, *p* < .001). Transparent proverbs versus opaque proverbs activated the left MTG.

**Table 4:** Anatomical regions that showed significant between‐group differences for figurative sentences

kE	*T*	Lat	Anatomical region	*x*	*y*	*z*	BA
Opaque proverbs > Transparent proverbs							
18	3.31	RH	Supramarginal gyrus	60	−38	28	40
34	3.52	RH	Precuneus	2	−60	52	7
Transparent proverbs > Opaque proverbs							
14	3.41	LH	Middle temporal gyrus	−60	−34	−4	21

L, left; R, right; H, hemisphere.

All areas reported were significant at *p* uncorrected ≤.001 (*T* ≥ 3.30), the spatial extent of activation (kE) was ≥10 voxels. Areas are presented with the stereotactic coordinates according to Talairach and Tournoux (*x*,* y*,* z*;1998), the cytoarchitectural designation according to Brodmann (BA), the maximum *T* value (*T*), and the extent (kE) of the activated clusters. In order to emphasize laterality effects, the hemispheric lateralization (Lat) is presented in a separate column.

## 4 DISCUSSION

In this study, we investigated the neural activation patterns associated with the reading of proverbs. We reported that different types of sentences activated distinct regions, supporting the hypothesis that transparency and literality are crucial in representing the neural basis of a metaphor. Indeed, we observed distinct effects of literality and transparency on brain activation.

### 4.1 Proverbs versus literal sentences

In our present study, compared with literal sentences, a significant activation pattern in the LH was found in response to proverbs. We observed robust LH activation for both transparent and opaque proverbs compared with literal sentences in well‐recognized semantic areas of the left IFG. The areas that were activated in response to these types of figurative sentences encompassed the classic semantic processing areas in the LH, including the left IFG and anterolateral temporal cortex (Oliveri et al., 2004; Rapp, Leube, Erb, Grodd, & Kircher, 2007). In line with our present findings, Bohrn, Altmann, and Jacobs (2012) reported that, although some RH activation was frequently observed in response to figurative language, the patterns of activation of the RH are less overlapping and more variable across studies than the activation of left frontotemporal regions. They additionally concluded that more studies will be needed to distinguish figurativity from other possible confounding factors, such as familiarity and syntactic complexity, and to break down further the various components of figurative language processing (Bohrn et al., 2012).

However, our present findings were not in agreement with other previous neuroimaging findings (Bottini et al., 1994; Zempleni et al., 2007). Bottini et al. (1994) investigated the cerebral activity in six healthy volunteers using positron emission tomography to test the hypothesis that the RH has a specific role in interpreting the figurative aspects of language, such as metaphors. The authors reported that the comprehension of metaphors was associated with several LH areas, including the basal frontal and prefrontal cortex, temporal pole, middle/inferior temporal gyri, precuneus, and parietal cortex. Additionally, several sites were found to be activated in the RH, including the precuneus, MTG, and prefrontal cortex. The authors concluded that the processing language involves widespread distributed bilateral systems and that the RH has a special role in the processing of metaphors. However, Bottini et al. (1994) compared the activity associated with the comprehension of metaphoric sentences with lexical‐decision tasks; thus, their findings may differ from ours in this study. More recently, Zempleni et al. (2007) reported in a comparison of figurative versus literal sentences that the former elicited activation in the bilateral IFG and bilateral MTG.

Our present findings are in agreement with previous studies. Taken together, these findings suggest that the RH theory of metaphor comprehension should be critically reevaluated (Duncan & Owen, 2000; Rapp et al., 2004; Stringaris, Medford, Giampietro, Brammer, & David, 2007; Wagner, Pare‐Blagoev, Clark, & Poldrack, 2001). Indeed, the reading metaphors, in contrast to the literal sentences, resulted in signal changes in the left lateral inferior frontal, inferior temporal, and posterior middle/inferior temporal gyri (Rapp et al., 2004). Stringaris et al. (2007) also reported that the activation of the left IFG was common to both nonmeaningful and metaphoric sentences, but not literal sentences. Our findings also corroborated previous findings that the left IFG mediates the retrieval and/or selection of semantic knowledge (Duncan & Owen, 2000). However, an alternative interpretation of this finding is that it may be attributable to an increased demand for control during semantic retrieval, irrespective of whether or not retrieval requires selection against competing representations, which has been suggested by Wagner et al. (Wagner et al., 2001).

### 4.2 Transparent proverbs versus opaque proverbs

The transparent proverbs versus opaque proverbs contrast further indicated that the opaque proverbs activated the SMG and right precuneus.

This finding is in contrast to the fMRI study of Zempleni and colleagues (Zempleni et al., 2007), which reported no activation in any brain regions, even at the relatively tolerant threshold of uncorrected *p *<* *.001. In contrast, previously reported divided visual field studies suggested that the RH is involved in ambiguous words comprehension (Faust & Chiarello, 1998), which supports the hypothesis that the RH may have some independent functions for difficult tasks. Alternatively, RH homologs may be activated to ‘help the LH, so more difficult linguistic stimuli may preferentially activate the RH.’ Almost all the studies that reported RH activation have used semantic relationships (Rapp et al., 2007, 2012). These findings supported theories that the RH is involved in the processing of distant relationships, which may be useful in both creative thought and problem solving.

In our present study, opaque proverbs activated the right SMG and right precuneus. The right SMG is generally thought to be involved in phonological word decisions, while the right precuneus has been shown to play an important role in episodic memory retrieval (Nyberg et al., 2000). A previous study reported that the precuneus was activated during source memory episodic retrieval (Lundstrom, Ingvar, & Petersson, 2005). According to a study of a similar design of Chinese idiom, the right precuneus was significantly activated upon the presentation of an opaque idiom, which is in line with our results (Yang et al., 2016). According to Mashal et al. (2005), the right precuneus was thought to be the main network for processing novel metaphor, and was considered to be unrelated to conventional metaphor. However, we suggest that the right precuneus is activated in relation to opaque proverbs with controlled familiarity, indicating that this region also participates in abstract language processing when the familiarity is controlled. Although only a few studies reported on the activation of the SMG under certain circumstances, it has been recently demonstrated that the right SMG is activated in relation to opaque idioms (vs. nonidiomatic phrases). Taken together with our study results, the right SMG may be involved in abstract language processing.

### 4.3 Limitations

Task demand, concreteness, and grammatical difficulty are known to affect the activation area. It is known that difficult metaphor activates left IFG rather than easy metaphor (Schmidt & Seger, 2009), but the control of difficulty was not complete in our present study. The concreteness of the proverb may be different from the literal sentence, which may be a confounding factor, but it was difficult to control because of the inherent differences between proverb and literal sentence (Lai, van Dam, Conant, Binder, & Desai, 2015). Finally, although the grammatical difficulty was not completely controlled, we tried to supplement it by setting the number of characters in each group to be similar.

## 5 CONCLUSIONS

Herein, we reported robust LH activation for Korean proverbs compared with literal sentences in semantic areas of the left IFG. Compared with transparent proverbs, opaque proverbs activated the right SMG and right precuneus. This study indicated that the transparency/opacity of proverbs is related to the site of activation. Opaque proverbs tend to have a distant semantic relationship, indicating thus that right SMG and precuneus may play a role in this process.

## CONFLICT OF INTEREST

None declared.

## AUTHOR CONTRIBUTION

Each author made a substantial contribution to the intellectual content of this manuscript and participated in the work to an extent sufficient to allow them to assume public responsibility for its content.
